# An Optimization Coverage Strategy for Wireless Sensor Network Nodes Based on Path Loss and False Alarm Probability

**DOI:** 10.3390/s25020396

**Published:** 2025-01-10

**Authors:** Jianing Guo, Yunshan Sun, Ting Liu, Yanqin Li, Teng Fei

**Affiliations:** School of Information Engineering, Tianjin University of Commerce, Tianjin 300134, China; 120220547@stu.tjcu.edu.cn (J.G.); sunyunshan@tjcu.edu.cn (Y.S.); liyanqin-2190@126.com (Y.L.); feiteng@tjcu.edu.cn (T.F.)

**Keywords:** WSN, coverage optimization problem, path loss, Neyman–Pearson criterion

## Abstract

In existing coverage challenges within wireless sensor networks, traditional sensor perception models often fail to accurately represent the true transmission characteristics of wireless signals. In more complex application scenarios such as warehousing, residential areas, etc., this may lead to a large gap between the expected effect of actual coverage and simulated coverage. Additionally, these models frequently neglect critical factors such as sensor failures and malfunctions, which can significantly affect signal detection. To address these limitations and enhance both network performance and longevity, this study introduces a perception model that incorporates path loss and false alarm probability. Based on this perception model, the optimization objective function of the WSN node optimization coverage problem is established, and then the intelligent optimization algorithm is used to solve the objective function and finally achieve the optimization coverage of sensor nodes. The study begins by deriving a logarithmic-based path loss model for wireless signals. It then employs the Neyman–Pearson criterion to formulate a maximum detection probability model under conditions where the cost function and prior probability are unknown, constraining the false alarm rate. Simulated experiments are conducted to assess the influence of various model parameters on detection probability, providing comparative analysis against traditional perception models. Ultimately, an optimization model for WSN coverage, based on combined detection probability, is developed and solved using an intelligent optimization algorithm. The experimental results indicate that the proposed model more accurately captures the signal transmission and detection characteristics of sensor nodes in WSNs. In the coverage area of the same size, the coverage of the model constructed in this paper is compared with the traditional 0/1 perception model and exponential decay perception model. The model can achieve full coverage of the area with only 50 nodes, while the exponential decay model requires 54 nodes, and the coverage of the 0/1 model is still less than 70% at 60 nodes. According to the simulation experiments, it can be basically proved that the WSN node optimization coverage strategy based on the proposed model provides an effective solution for improving network performance and extending network lifespan.

## 1. Introduction

Wireless sensor networks (WSNs) are distributed networks configured in a self-organizing, multi-hop architecture [1,2], comprising stationary or mobile sensor nodes. These nodes are capable of sensing information, are equipped with storage and communication functionalities, and can collaboratively perceive, collect, process, and transmit data about objects within their coverage area, ultimately relaying this information to the network owner [3]. Collectively, WSNs, communication technology, and computer technology form the foundational pillars of information technology. With the rapid advancement and integration of the Internet of Things (IoT), artificial intelligence (AI), and big data, WSNs have garnered significant attention from the global academic community [4,5,6,7], being regarded as a core and essential technology at the forefront of sensing. He et al. [8] studied the construction of a UAV-based recording base station based on WSN, which is expected to be an important component of future intelligent transportation systems. Gutierrez et al. [9] studied the application of low-cost WSNs in crop soil detection, providing a new scheme for humidity detection in smart farms. Ramya et al. [10] discussed the influence of the WSN cluster head selection strategy on the Internet of Things and analyzed the influence of different strategies on network devices and device life. Driven by extensive data requirements and widespread detection needs, the primary role of WSNs has evolved from localized sensing to large-scale connectivity, finding applications across various societal domains, including military security, healthcare, transportation, industry, and disaster prevention [11,12].

The WSN coverage problem pertains to the strategic deployment of numerous sensor nodes within a network, where node coverage is critical for ensuring network lifespan, quality, performance, and resource efficiency [13]. As a result, optimizing WSN node coverage has emerged as a central focus of research. The process of addressing this problem can typically be segmented into four stages. Initially, the type of coverage necessary for the specific application scenario should be identified to determine the constraints and optimization objectives. Subsequently, a sensor perception model should be developed to mathematically represent the sensor’s perception characteristics. The third step involves considering relevant network performance parameters and constructing an evaluation function based on the defined optimization objectives. Finally, an appropriate solution method should be employed to optimize node placement [14,15].

The sensor node perception model serves as a fundamental component for addressing the WSN node coverage optimization problem. An evaluation function based on a more objective perception model can enhance the practical applicability of an optimized coverage strategy, thereby improving network performance, extending network lifespan, and minimizing resource wastage. Traditional perception models primarily include the 0/1 perception model, the attenuation perception model, and the probabilistic perception model [16,17,18]. The 0/1 perception model is highly idealized, neglecting real-world considerations such as signal transmission attenuation and variations in sensor performance, focusing solely on the sensor’s perception radius. Conversely, the attenuation perception model incorporates the proportional relationship between wireless signal transmission attenuation and relative distance, constructing an attenuation-based model according to transmission distance. In the context of WSN node coverage optimization research, developing a mathematical model that objectively captures sensor perception characteristics is crucial.

In recent years, the research on the WSN node optimization coverage problem mainly focuses on the optimization of multi-network parameters and the improvement of optimization algorithms. With the application and development of intelligent optimization algorithms, there are many papers that improve the optimization algorithm to solve the coverage problem better. Yi et al. [19] improved the greedy strategy to solve the underwater target optimization coverage problem based on the 0/1 perception model by combining the depth-first search mechanism. Simulation experiments verified that its improved scheme could effectively improve network coverage and connectivity performance compared with the ACO-based deployment algorithm. Wang et al. [20] proposed an evolutionary algorithm based on an elite differential feedback strategy to solve the area optimization coverage problem based on the 0/1 perception model. Through simulation experiments, this algorithm can solve the deployment scheme with higher coverage than the original evolutionary algorithm and several improved algorithms. In order to improve the comprehensive performance of the network, some scholars set up a multi-objective optimization model from multiple network performance indicators. Li et al. [21] established a multi-objective optimization model of coverage rate, coverage efficiency, redundancy coverage rate, and moving distance, and the model was solved by an improved sand cat swarm optimization algorithm. Simulation experiments show that the scheme can obtain better distribution characteristics and network performance. Jebi et al. [22] established an optimization objective function based on coverage, network connectivity, the selection of capable connection points, and the number of mobile nodes, and simulation experiments verified that the scheme had certain application value in improving network life and optimizing network energy consumption. Through the research and analysis of a large number of studies in recent years, the existing research on WSN node optimization coverage mainly has the following defects:

- Most of the research focuses on the improvement of optimization algorithms, but the basic model of network coverage is not studied and discussed much.
- The sensor based on the 0/1 perception model is often regarded as an independent detection unit, but in the actual WSN, the work of multiple sensors is not completely independent, and the joint perception function of multiple sensors should be considered.
- In the wider application scenarios of WSN, there is often a lot of interference, the sensor as a sensing instrument has the possibility of misjudgment or failure, and the existing model lacks response to such problems.

Some researchers have begun to try to solve the situation where the actual coverage is inconsistent with the coverage expectation based on the idealized model. For example, Ali et al. [23] introduced a dynamic threshold detection probability (TDP) that decays with distance. Comparing the exponential decay probability of the target location to the dynamic TDP rather than the traditional static TDP and adding the signal-to-noise ratio to simulate the signal transmission in a real environment, Yang et al. [24] studied the change law of joint perception of multiple sensors under different surround structures under the attenuation perception model. Jin et al. [25] studied the problem of permanent target coverage under the constraint of non-uniform distribution of sensor detection energy. This research is motivated by the lack of research on the perception model that can reflect the signal attenuation and signal misjudgment in real sensor operation.

In this research, we propose that the sensor node’s perception of a target point encompasses both signal transmission and detection processes. To construct a perception model that accurately represents the principles of wireless signal attenuation and detection and provides a practically viable solution for the WSN node coverage optimization problem, this study presents an optimized coverage strategy based on path attenuation and false alarm probability. The main contributions of this paper are as follows:

- By investigating the characteristics of wireless signal transmission and detection, we account for the multipath characteristics of non-line-of-sight transmission and the impact of false alarm and missed detection rates in signal detection. We construct a probabilistic perception model and analyze the influence of key parameters on this model.
- Recognizing that, in WSNs, the perception of any point typically results from the cooperative functioning of multiple sensors rather than a single sensor, we develop an evaluation function for network coverage based on the proposed model and multi-sensor joint perception relationships.

The remainder of this paper is structured as follows: Section 2 reviews and evaluates various traditional perception models and detection theories. Section 3 derives a perception model that maximizes detection probability using a wireless signal path attenuation model and the Neyman–Pearson criterion, and simulation experiments are used to analyze the influence of model parameters on perception probability. Section 4 constructs a joint perception model for multiple sensors in the network and analyzes the effect on the effective coverage area given a fixed number of nodes in a full coverage scenario. Section 5 presents the solution to the model through simulation experiments and compares it with traditional perception models. Section 6 concludes the study with a brief summary.

## 2. Research Background

This section presents an overview of existing research on WSN node coverage challenges and signal detection, emphasizing perception models related to WSN node coverage and traditional detection theories in signal detection.

### 2.1. The Types of WSN Node Coverage Problems

The types of WSN node coverage problems can be classified in several ways. According to different detection purposes, they can be divided into target coverage, fence coverage, and area coverage.

Target coverage’s main function is to detect one or more groups of target points that have been specified in the area, requiring that the deployment of sensors can effectively cover all discrete detected points [26,27]. Fence coverage focuses on monitoring the crossing behavior of unknown moving points [28,29]. It is mainly used in security defense and protection scenarios to monitor the crossing behavior of moving points from one side of the boundary line to the other side. Area coverage involves covering the whole target area to a certain extent to meet the needs of various and large amounts of data collection in the big data environment [30,31].

Different deployment modes can be divided into random coverage and deterministic coverage. Random coverage is usually applicable to complex or harsh situations that are difficult to intervene in for human beings, or situations where the relevant information about the coverage area is unknown and difficult to plan [32]. The sensor is randomly distributed in the area by a certain sprinkling mode. In contrast, deterministic coverage refers to the known coverage of the location and the number of nodes deployed [33].

According to whether the network node has a certain mobile ability in operation, it can be divided into static network, dynamic network, and hybrid network [34,35].

### 2.2. Sensor Node Perception Model

A sensor’s perception model is a mathematical representation of its sensing capabilities, illustrating the relationship between the node’s sensing range, perception characteristics, intensity features, and the detection target. This model serves as the foundational basis for addressing sensor coverage problems. The selection of a perception model significantly impacts whether the network configuration meets the expected objectives. Choosing an appropriate perception model tailored to different coverage environments and specific requirements can enhance the practical utility of network planning. Common perception models include the 0/1 perception model, the attenuation perception model, and the probabilistic perception model.

Assume there are n sensor nodes in the network, represented by the sensor set $S=\left\{s_{1},s_{2},\cdots ,s_{n}\right\}$, with the coordinates of any node $s_{i}$ denoted as $\left(x_{i},y_{i}\right)$. The entire coverage area is discretized into multiple detection nodes with unknown information, represented by the set $T=\left\{t_{1},t_{2},\cdots ,t_{m}\right\}$, where the coordinates of any detection node $t_{j}$ are $\left(x_{j},y_{j}\right)$.

During operation, a sensor undergoes two primary signal transmission processes: detecting the target and communicating with other nodes. Each sensor is assumed to have a sensing radius ($r_{s}$) and a communication radius ($r_{c}$). To maintain network connectivity, it is typically established that $r_{c}\geq 2r_{s}$ [36,37].

#### 2.2.1. 0/1 Perception Model

The 0/1 perception model, also known as the deterministic perception model or Boolean model, is one of the most traditional approaches in perception modeling. This model disregards practical considerations such as signal transmission attenuation and variations in sensor performance, resulting in a simplified, idealized representation [38,39,40]. Under this model, the probability of a sensor $s_{i}$ detecting a target $t_{j}$ can be expressed as

(1) $$p_{\mathrm{cov}}(s_{i},t_{j})=\left\{\begin{array}{l} \;1\;,\;d_{ij}\leq r_{s}\\ \;0\;,\;r_{s}<d_{ij} \end{array}\right.$$

where $d_{ij}$ is the Euclidean distance from sensor $s_{i}$ to detection point $t_{j}$.

Due to its intuitive nature and the simplicity of the perception relationships between multiple nodes, this model is frequently utilized as the fundamental perception model in research. Studies [41,42,43] have developed objective functions for WSN coverage optimization based on this model, achieving higher coverage rates for target areas through various enhanced optimization algorithms.

#### 2.2.2. Exponential Decay Model

The exponential decay model is an attenuation model that leverages the properties of exponential functions. This model can be categorized into two types. The first type assumes that detection within the sensor’s sensing radius is certain, while beyond this radius, the detection probability decays exponentially [44,45]. The second type assumes that the detection probability decays exponentially within the sensing radius, and detection becomes impossible beyond this radius [23,46]. The formulas for these two perception models are as follows:

(2) $$p_{\mathrm{cov}}(s_{i},t_{j})=\left\{\begin{array}{cc} 1 & ,\;d_{ij}\leq r_{s}\\ e^{-C(d_{ij}-r_{s})} & ,\;r_{s}<d_{ij} \end{array}\right.$$

(3) $$p_{\mathrm{cov}}(s_{i},t_{j})=\left\{\begin{array}{cc} \;e^{-Cd_{ij}} & ,\;d_{ij}\leq r_{s}\\ \;0\; & ,\;r_{s}<d_{ij} \end{array}\right.$$

where C is a constant and serves as the sensing decay factor.

### 2.3. Classical Signal Detection Criteria

In wireless detection systems, signals are often affected by various types of noise and signal interference during transmission, leading to waveform distortion. The receiver must then interpret the original signal based on the distorted waveform it receives. Detection theory provides a theoretical framework for determining the presence or type of a signal using limited observational data in environments with noise and interference. Due to signal distortion and receiver influences, detection outcomes may not always be accurate; instead, the objective is to make decisions that meet specific criteria as closely as possible. Common decision-making criteria include the Bayes criterion, the minimax criterion, and the Neyman–Pearson criterion.

For the transmitter, there are two hypotheses: the presence of a transmitted signal (denoted $H_{1}$) and the absence of a transmitted signal (denoted as $H_{0}$). For the receiver, the two possible decisions are that the signal is present (denoted as $D_{1}$) or absent (denoted as $D_{0}$). These combinations lead to four possible decision outcomes, each with associated probabilities. These outcomes are represented as $p(D_{0}|H_{0})$, $p(D_{1}|H_{1})$, $p(D_{1}|H_{0})$, and $p(D_{0}|H_{1})$. The first two represent correct detections and are known as the detection probability. In contrast, if $H_{1}$ occurs but $D_{0}$ is decided, this is referred to as a false alarm or Type I error. A false alarm means that the sending end does not send a signal, and the receiving end mistakenly determines that there is a signal. Conversely, if $H_{0}$ occurs but $D_{1}$ is decided, this is known as a miss or Type II error. A miss means that the sender sends a signal, but the receiver does not decide that the signal exists. Under the Bayes criterion, the hypothesis with the higher posterior probability $p(H_{0}|x)$ and $p(H_{1}|x)$), given the observation *x*, is chosen as the decision outcome. The decision rule can be expressed as

(4) $$\frac{p(H_{1}|x)}{p(H_{0}|x)}\;\begin{array}{c} \overset{H_{1}}{>}\\ \underset{H_{0}}{<} \end{array}\;1$$

By substituting Bayes’ formula to express the posterior probabilities, the decision rule can be reformulated in terms of the likelihood function and prior probability

(5) $$\frac{f(x|H_{1})}{f(x|H_{0})}\;\begin{array}{c} \overset{H_{1}}{>}\\ \underset{H_{0}}{<} \end{array}\;\frac{p(H_{1})}{p(H_{0})}=l(x)$$

where l(x) is the likelihood ratio.

Under the Bayes criterion, both the prior probability and cost function must be known. When the prior probability is not available, the minimax criterion can be employed as an alternative. This criterion identifies a prior probability that minimizes the maximum risk, which is then substituted into the Bayes decision formula to derive the minimax decision rule.

For detection scenarios involving sensors, radar, and similar applications, specifying a cost function and prior probability can be challenging, necessitating the use of the Neyman–Pearson criterion. This criterion assumes that one type of error is of greater importance, thereby strictly constraining the probability of that error while determining a decision threshold that minimizes the probability of other errors within this constraint. The Neyman–Pearson criterion aims to maximize the detection probability and minimize the miss probability, given a predetermined false alarm probability.

## 3. Perception Model Based on Path Loss and False Alarm Probability

This section presents the perception model we developed, which incorporates path loss and false alarm probability. Using MATLAB 2018b simulations, we analyze the influence of various parameters on the perception probability curve of the proposed model and compare it with the perception probability curves of traditional models. Additionally, to address the requirements of the coverage optimization problem, we construct an objective function based on the joint perception probability of multiple sensors.

### 3.1. Model Derivation

The perception process of sensor nodes in a WSN can be described as follows: a target point emits a wireless signal, which reaches the sensor nodes in the network via line-of-sight or non-line-of-sight transmission. The sensor nodes then evaluate the received signal to determine whether it originated from the target point’s location.

For the propagation process of the signal, it is obvious that the power of the signal will be attenuated when the distance between the sending and receiving ends is far away, and it is difficult to reasonably reflect the law of this attenuation in the common perception model. Considering that WSN is often used in complex environments with a large number of non-line-of-sight transmissions, this paper adopts the logarithmic path attenuation model to determine the signal attenuation.

The attenuation model of non-line-of-sight transmission should be derived from the free space attenuation model. Signal transmission in free space is often considered to be a signal directly from the sending point to the receiving point, and the attenuation power of the received signal strength is

(6) $$P_{r}(d)=\frac{P_{t}G_{t}G_{r}\lambda^{2}}{{\left(4\pi \right)}^{2}d^{2}L}$$

where $P_{r}$ and $P_{t}$ represent the received and transmitted power, respectively. $G_{t}$ and $G_{r}$ denote the antenna gains at the transmitter and receiver, respectively. λ is the wavelength of the transmitted signal, d is the distance between the transmitter and receiver, and L is the system loss factor attributed to hardware inefficiencies. Converting the equation into a gain-based form for path loss results in

(7) $$\begin{array}{ll} PL_{F}(d)[dB] & =10\mathrm{lg}\left(\frac{P_{t}}{P_{r}}\right)=-10\mathrm{lg}\left(\frac{G_{t}G_{r}\lambda^{2}}{{\left(4\pi \right)}^{2}d^{2}L}\right)\\ & =-10\mathrm{lg}G_{t}G_{r}+20\mathrm{lg}\frac{4\pi }{\lambda }+20\mathrm{lg}d+10\mathrm{lg}L \end{array}$$

In the presence of obstructions, a path loss exponent η, which varies depending on the environment, can be introduced to modify the equation, resulting in a path loss model suitable for obstructed conditions

(8) $$PL_{LD}(d)[dB]=PL_{F}(d_{0})+10\eta \mathrm{lg}\left(\frac{d}{d_{0}}\right)$$

where $d_{0}$ represents the reference distance, which is determined by the network characteristics. For macrocell systems with a radius greater than 10 km, $d_{0}$ is typically set to 1 km. For cellular systems with a radius of approximately 1 km, $d_{0}$ is usually set to 100 m. For microcell systems with an even smaller radius, $d_{0}$ is often set to 10 m. The path loss exponent η depends on the complexity of the propagation environment and generally ranges from 2 to 6. In free-space propagation, η is 2, and it increases as the number of obstacles increases. Table 1 provides path loss exponents for various environments.

Formula (8) is the logarithmic path attenuation model, which can be used to calculate the attenuation power of wireless signal after non-line-of-sight transmission with attenuation or occlusion. By changing parameter η, scenes with different occlusion degrees can be described. The received power at a distance d can be expressed as

(9) $$P_{r}(d)[dB]=P_{t}-PL_{LD}(d)[dB]=P_{t}-PL_{F}(d_{0})-10\eta \mathrm{lg}\left(\frac{d}{d_{0}}\right)$$

Formula (9) can reflect the attenuation of the signal in the transmission process. However, in the real detection environment, the signal received by the sensor will receive a certain amount of noise in addition to the useful signal, which will make it more difficult for the sensor to judge the signal, and there will be wrong decisions. At this time, it is necessary to further consider the impact of signal detection on the perception probability. For signal detection criteria with unknown parameters, the Bayes criterion is used when the prior probability and cost function of signal occurrence are known, while the maximin criterion is used if the prior probability is unknown. For the wireless signal detection problem considered in this paper, the prior probability of signal occurrence and the cost function of error decision are both unknown. Therefore, the Neyman–Pearson criterion is adopted here to limit the probability of false alarm error, determine the decision threshold that can minimize the probability of missing alarm, and then obtain the decision method that maximizes the detection probability.

For a signal transmission point in the network, the observation $y_{ti}$ at a sensor node located at a distance d is given by

(10) $$y_{ti}=\left\{\begin{array}{l} \;P_{r}+n_{i},\;H_{1}\;\\ n_{i},\;\;\;\;\;\;\;\;\;\;H_{0} \end{array}\right.$$

where $H_{1}$ and $H_{0}$ represent the cases of target presence and target absence, respectively. $n_{i}$ denotes Gaussian white noise with a mean of 0 and variance $\sigma^{2}$. Therefore, the distribution of the observation $y_{ti}$ is

(11) $$\left\{\begin{array}{l} H_{0}:\;y_{ti}\sim N\left(0,\sigma^{2}\right)\\ H_{1}:\;y_{ti}\sim N\left(P_{r},\sigma^{2}\right) \end{array}\right.$$

According to detection theory, the detection probability in state $H_{0}$ is given by

(12) $$p\left(y_{ti}\left|H_{0}\right.\right)=\frac{1}{\sqrt{2\pi \sigma^{2}}}\exp\left[-\frac{y_{ti}^{2}}{2\sigma^{2}}\right]$$

Similarly, the detection probability in state $H_{1}$ is given by

(13) $$p\left(y_{ti}\left|H_{1}\right.\right)=\frac{1}{\sqrt{2\pi \sigma^{2}}}\exp\left[-\frac{1}{2\sigma^{2}}{\left(y_{ti}-P_{r}\right)}^{2}\right]$$

From Equations (18) and (19), the likelihood ratio $l_{i}(y_{ti})$ is given by

(14) $$l_{i}(y_{ti})=\frac{p\left(y_{ti}\left|H_{1}\right.\right)}{p\left(y_{ti}\left|H_{0}\right.\right)}=\frac{\frac{1}{\sqrt{2\pi \sigma^{2}}}\exp\left[-\frac{1}{2\sigma^{2}}{\left(y_{ti}-P_{r}\right)}^{2}\right]}{\frac{1}{\sqrt{2\pi \sigma^{2}}}\exp\left[-\frac{y_{ti}^{2}}{2\sigma^{2}}\right]}=\exp\left(\frac{1}{\sigma^{2}}y_{ti}P_{r}-\frac{1}{2\sigma^{2}}P_{r}^{2}\right)$$

The likelihood ratio test is given by

(15) $$\exp\left(\frac{1}{\sigma^{2}}y_{ti}P_{r}-\frac{1}{2\sigma^{2}}P_{r}^{2}\right)\;\begin{array}{c} \overset{H_{1}}{>}\\ \underset{H_{0}}{<} \end{array}\;l_{i}(y_{ti})$$

Converting to logarithmic form, we have

(16) $$\frac{1}{\sigma^{2}}y_{ti}P_{r}-\frac{1}{2\sigma^{2}}P_{r}^{2}\;\begin{array}{c} \overset{H_{1}}{>}\\ \underset{H_{0}}{<} \end{array}\;\ln l_{i}(y_{ti})$$

After further simplification, we obtain

(17) $$\underset{g}{\underset{︸}{P_{r}y_{ti}}}\;\begin{array}{c} \overset{H_{1}}{>}\\ \underset{H_{0}}{<} \end{array}\;\underset{h}{\underset{︸}{\sigma^{2}\ln l_{i}(y_{ti})+\frac{1}{2}P_{r}^{2}}}$$

Let the two sides of the decision rule be represented as g and h, respectively. For a known set of nodes, all terms on the right side (h) except for the likelihood ratio $l_{i}(y_{ti})$ are fixed and known. Once a decision is made, yielding a satisfactory statistic g, we can further determine $y_{ti}$. Based on the properties of the mean and variance of Gaussian random variables, the distribution of the statistic g under $H_{1}$ and $H_{0}$ is given by

(18) $$\left\{\begin{array}{l} H_{0}:\;g\sim N\left(0,P_{r}^{2}\sigma^{2}\right)\\ H_{1}:\;g\sim N\left(P_{r}^{2},P_{r}^{2}\sigma^{2}\right) \end{array}\right.$$

Let the mean and variance of the statistic g be represented as

(19) $$\left\{\begin{array}{l} \mu_{g}=P_{r}^{2}\\ \sigma_{g}^{2}=P_{r}^{2}\sigma^{2} \end{array}\right.$$

Then, the false alarm rate $p_{F}$ and detection probability $p_{D}$ based on the statistics are given by

(20) $$\begin{array}{l} p_{F}=p\left(g>h\left|H_{0}\right.\right)=1-p\left(g\leq h\left|H_{0}\right.\right)=1-p\left(\frac{g}{\sigma_{g}}\leq \frac{h}{\sigma_{g}}\left|H_{0}\right.\right) \end{array}$$

(21) $$\begin{array}{l} p_{D}=p\left(g>h\left|H_{1}\right.\right)=1-p\left(g\leq h\left|H_{1}\right.\right)=1-p\left(\frac{g-\mu_{g}}{\sigma_{g}}\leq \frac{h-\mu_{g}}{\sigma_{g}}\left|H_{1}\right.\right) \end{array}$$

Clearly, $\frac{h}{\sigma_{g}}$ and $\frac{h-\mu_{g}}{\sigma_{g}}$ in the equation follow standard Gaussian distributions under states $H_{0}$ and $H_{1}$, respectively. Let Φ(⋅) denote the cumulative distribution function of the standard Gaussian distribution, defined as

(22) $$\Phi (z)=p\left(X\leq z\right)=\frac{1}{\sqrt{2\pi }}\int_{-\infty }^{z}\exp\left(-\frac{\tau^{2}}{2}\right)d\tau$$

Then, Equations (26) and (27) can be rewritten as

(23) $$p_{F}=1-\Phi \left(\frac{h}{\sigma_{g}}\right)$$

(24) $$p_{D}=1-\Phi \left(\frac{h-\mu_{g}}{\sigma_{g}}\right)=1-\Phi \left(\frac{h}{\sigma_{g}}-\frac{\mu_{g}}{\sigma_{g}}\right)$$

According to the Neyman–Pearson criterion, let the acceptable false alarm rate be $p_{F}=\alpha$. Substituting this into Equation (24) yields

(25) $$1-\Phi \left(\frac{h}{\sigma_{g}}\right)=\alpha \Rightarrow \frac{h}{\sigma_{g}}=\Phi^{-1}\left(1-\alpha \right)$$

According to Equation (25), we have

(26) $$\frac{\mu_{g}}{\sigma_{g}}=\frac{P_{r}^{2}}{P_{r}\sigma }=\frac{P_{r}}{\sigma }$$

By combining Equations (25)–(27), the perception model based on path loss and maximized detection probability can be expressed as

(27) $$\begin{array}{ll} p_{D} & =1-\Phi \left[\Phi^{-1}\left(1-\alpha \right)-\frac{P_{r}}{\sigma }\right]\\ & =1-\Phi \left[\Phi^{-1}\left(1-\alpha \right)-\frac{P_{t}G_{t}G_{r}\lambda^{2}}{\sigma {\left(4\pi \right)}^{2}L}\cdot \frac{d_{0}^{\eta -2}}{d^{\eta }}\right] \end{array}$$

The formula is based on the attenuation of wireless signals under real non-line-of-sight propagation and rarely combines the signal detection situation to construct the calculation formula of perception probability. In this model, the possibility of sensor misjudgment and failure can be taken into account at the same time of signal attenuation.

### 3.2. Maximum Sensing Radius

**Figure 1:**
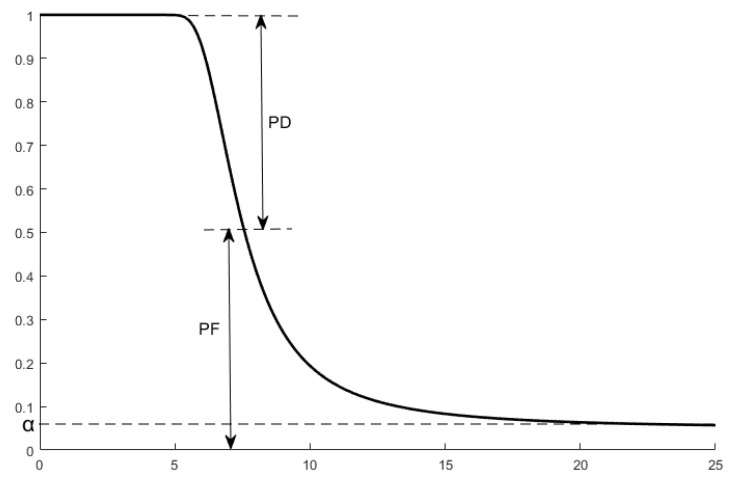
The relationship between $p_{D}$ and $p_{F}$.

Since the concept of false alarm probability exists in signal detection, the actual detection probability $p_{D\;actual}$ of an event is correct detection probability $p_{D}$ plus false alarm probability $p_{F}$. However, the maximum acceptable false alarm probability is defined as α in the Neyman–Pearson criterion. Therefore, as the distance between the target and the sensor increases, the correct detection probability $p_{F}$ of the signal will not decay to 0 but to α. The relationship between $p_{D}$ and $p_{F}$ is shown in Figure 1.

Obviously, in this case, it is unrealistic to calculate the detection probability of the target point very far from the sensor by Equation (27), which is not 0. Therefore, it is necessary to define a minimum detectable received power $P_{r0}$. When the signal is far from the sensor, the signal power is too small to be detected at all, and the undetectable distance threshold is the maximum sensing radius R. The relation between $P_{r0}$ and R can be determined by Equation (10)

(28) $$P_{r0}[dB]=P_{t}-PL_{F}(d_{0})-10\eta \mathrm{lg}\left(\frac{R}{d_{0}}\right)$$

After simplifying and converting to a non-dB form, the expression for R is given by

(29) $$R=\sqrt[{\eta }]{\frac{P_{t}}{P_{r0}}\cdot \frac{G_{t}G_{r}\lambda^{2}d_{0}^{\eta -2}}{{\left(4\pi \right)}^{2}L}}$$

**Figure 2:**
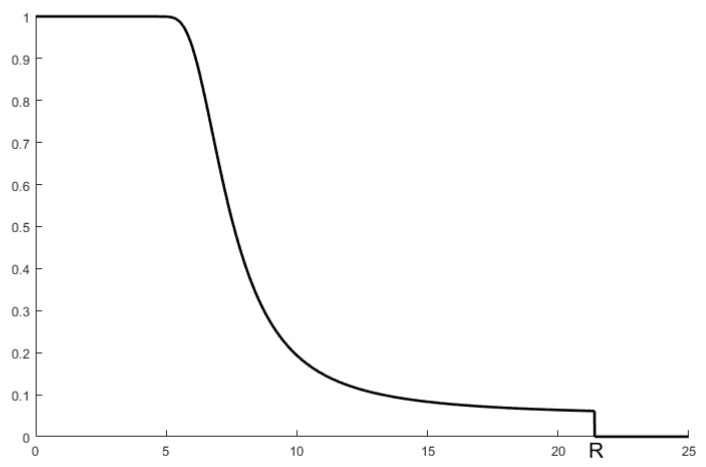
The improved perceived probability change curve.

The maximum perception radius is introduced, and the perception probability decreases to 0 when the distance is greater than R. The perception probability calculation Formula (27) is modified as follows, and the change curve is shown in Figure 2.

(30) $$p_{D}=\left\{\begin{array}{c} 1-\Phi \left[\Phi^{-1}\left(1-\alpha \right)-\frac{P_{t}G_{t}G_{r}\lambda^{2}}{\sigma {\left(4\pi \right)}^{2}L}\cdot \frac{d_{0}^{\eta -2}}{d^{\eta }}\right],\;d\leq R\\ \;\;\;\;\;\;\;\;0\;\;\;\;\;\;\;,\;d>R \end{array}\right.$$

### 3.3. Parameter Influence

In Equation (28), the parameters that primarily influence the detection probability curve include the false alarm rate α, variance σ, and path loss coefficient η. With the other parameters held constant, the detection probability curves for these three parameters at different values are plotted using MATLAB. The parameter settings are provided in Table 2.

**Figure 3:**
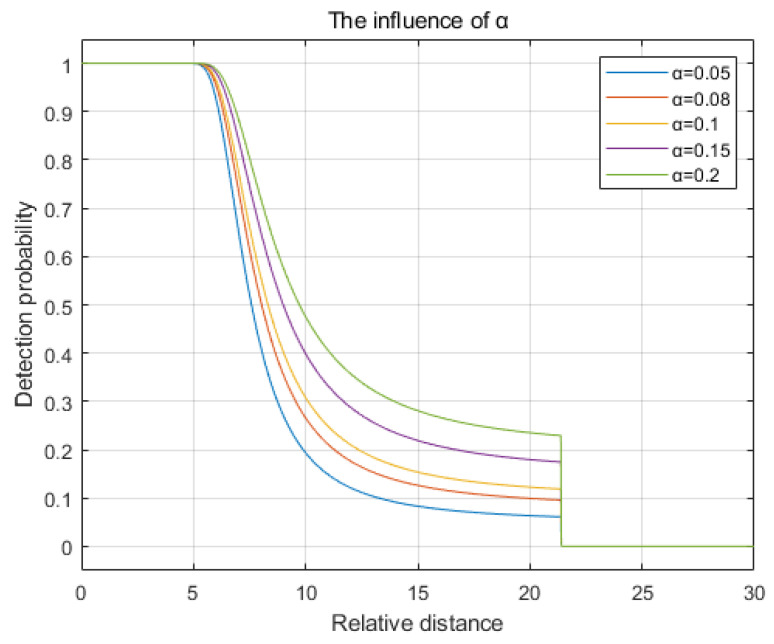
The influence of the false alarm rate α.

The acceptable false alarm rate α is set to 0.05, 0.08, 0.1, 0.15, and 0.2. The curve depicting detection probability as a function of relative distance is illustrated in Figure 3. It can be observed from the figure that as the false alarm rate α increases, the rate of decay in the detection probability slows, and the convergence value of the detection probability rises. However, the sensing radius remains unaffected.

**Figure 4:**
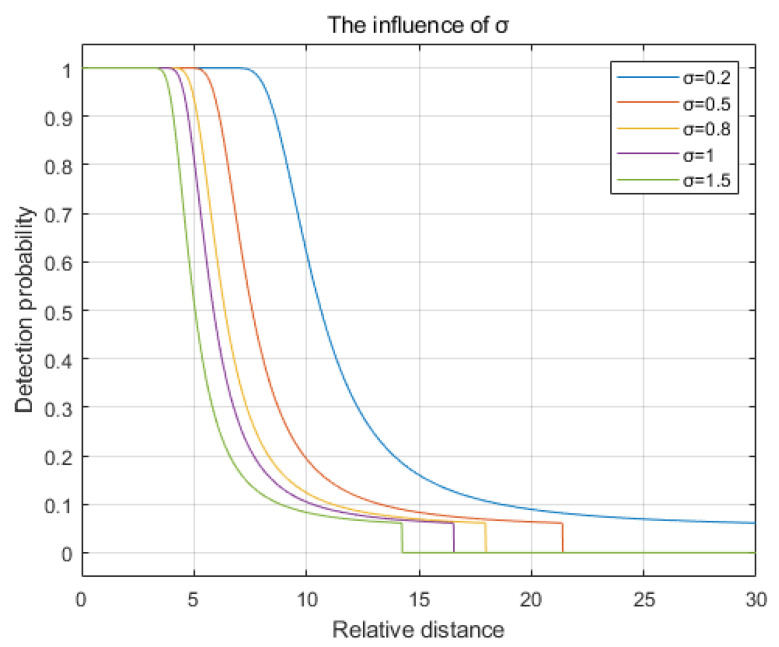
The influence of the variance σ.

The variance σ to 0.2, 0.5, 0.8, 1, and 1.5. The comparison chart illustrating the changes in detection probability is shown in Figure 4. As observed from the figure, as the variance σ increases, the rate of decay in detection probability accelerates, the decay starts earlier, and the sensing radius decreases.

**Figure 5:**
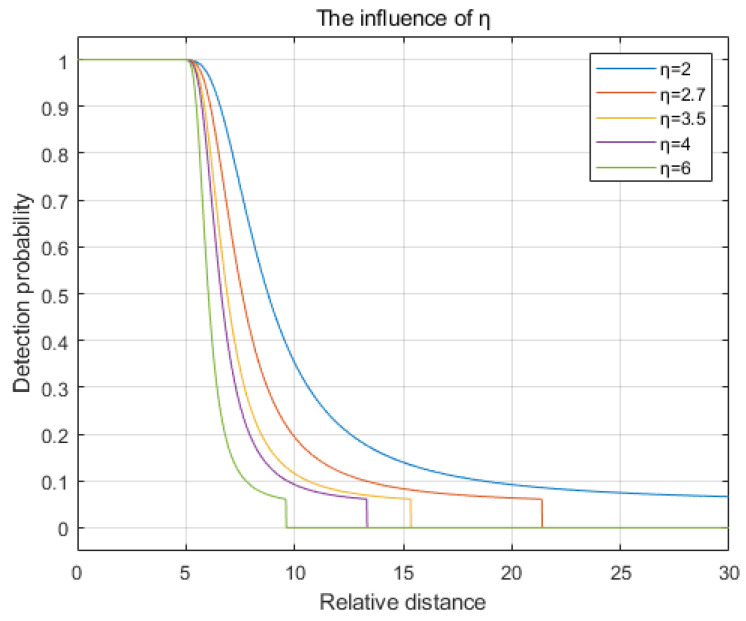
The influence of the path loss coefficient η.

The path loss coefficient η is set to values of 2, 2.7, 3.5, 4, and 6. The comparison chart illustrating the changes in detection probability is shown in Figure 5. As observed in the figure, with an increase in the number of obstacles, represented by a higher path loss coefficient η, the rate of decay in detection probability accelerates, and the detection radius decreases.

### 3.4. Differences Between Perception Models

**Figure 6:**
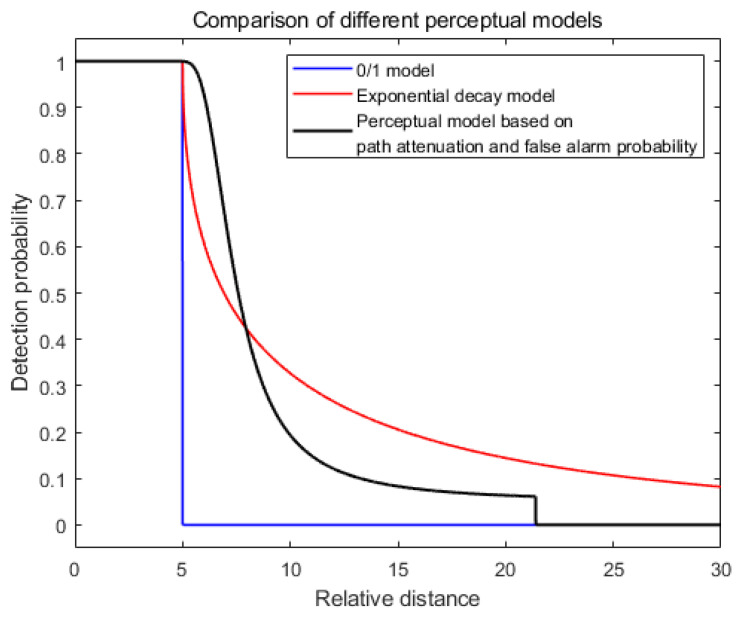
Comparison of different perceptual models.

Setting the parameters for the proposed perception model based on path loss and false alarm probability, as outlined in Table 2, the deterministic sensing radius R for the 0/1 model and the attenuation model is defined as the maximum distance where the detection probability has not decayed (set to 5 under the parameters in Table 2). The detection probability curves for the three models are shown in Figure 6. As observed in the figure, the proposed model achieves a larger sensing range compared to the 0/1 model, and its attenuation pattern more accurately reflects actual detection conditions compared to the exponential decay model.

## 4. Analysis of WSN Node Coverage Problem Based on the Probabilistic Model

This section primarily examines the WSN node coverage problem using the perception model derived above. It involves developing an effective coverage rate model for an area based on joint perception probability and analyzing the impact of different models on the number of nodes required for full coverage.

### 4.1. Coverage Rate Model Based on Joint Perception Probability

In a WSN, the sensing of any given point is typically the result of combined detection by multiple nodes rather than a single sensor. Therefore, for a known WSN, the miss probability $p_{miss}(S,t_{j})$ at a target point $t_{j}$ is given by

(31) $$p_{miss}(S,t_{j})=\prod_{i=1}^{n}\left[1-p_{\mathrm{cov}}(s_{i},t_{j})\right]$$

where, $p_{\mathrm{cov}}(s_{i},t_{j})$ is the perception probability of sensor $s_{i}$ for target point $t_{j}$, obtained from Equation (28)

(32) $$p_{\mathrm{cov}}(s_{i},t_{j})=p_{D}(d_{ij})=1-\Phi \left[\Phi^{-1}\left(1-\alpha \right)-\frac{P_{t}G_{t}G_{r}\lambda^{2}}{\sigma {\left(4\pi \right)}^{2}L}\cdot \frac{d_{0}^{\eta -2}}{d_{ij}^{\eta }}\right]$$

The coverage rate $p_{\mathrm{cov}}(S,t_{j})$ of the entire network for point $t_{j}$ is given by

(33) $$p_{\mathrm{cov}}(S,t_{j})=1-p_{miss}(S,t_{j})=1-\prod_{i=1}^{n}\left[1-p_{\mathrm{cov}}(s_{i},t_{j})\right]$$

For the 0/1 perception model, when $p_{\mathrm{cov}}(S,t_{j})=1$, the point is considered covered. However, for perception models with attenuation, the detection probability of a node decreases as the distance from the sensor increases, resulting in an attenuation trend in the perception characteristic curve, as opposed to the rectangular characteristic curve of the 0/1 model. In these models, a single sensor node cannot guarantee definitive detection of all targets within its sensing range, making it necessary to consider the joint perception of multiple nodes for a target point. When the joint perception probability at a given point reaches a predefined threshold $P_{th}$, the network is considered to have satisfactory perception capability for that point. Thus, the coverage rate for target $t_{j}$ should satisfy

(34) $$p_{\mathrm{cov}}(S,t_{j})=1-\prod_{i=1}^{n}\left[1-p_{\mathrm{cov}}(s_{i},t_{j})\right]\geq \varepsilon$$

The area coverage rate is a fundamental metric for WSNs, reflecting the network’s coverage level over the target area and serving as a primary evaluation standard for the network’s capability to perceive the physical world. This metric is defined as the ratio of the total area effectively covered by sensors to the area of the entire region. By discretizing the deployment area A and assuming its length and width are a and b, with a total of a×b detection nodes, the expression for the effective area coverage rate $C_{r}$ based on the joint perception probability in Equation (34) is given by

(35) $$C_{r}=\frac{\sum_{j=1}^{a\times b}\left\{p_{\mathrm{cov}}|p_{\mathrm{cov}}(S,t_{j})\geq \varepsilon \right\}}{a\times b}$$

The objective of WSN node optimization coverage is to maximize the area coverage rate under a limited number of sensors, expressed as $\max C_{r}$.

### 4.2. Computational Complexity

All discrete grids of the region need to be traversed to calculate the area coverage based on Equation (35), and there are n sensor nodes in the network. The perception probability of a certain point in the matrix must traverse all the sensors, calculate the perception probability of each sensor to the point, and compute the product. First, it is necessary to calculate the distance between the point and the sensor, and the time cost of this step is O(n). Secondly, it is necessary to determine the coverage relationship of the sensor to the point, and the time cost is O(n). Then, the perception probability of the sensor with coverage relationship is calculated, and the time cost is $O(n^{\prime })$, $\left(n^{\prime }\leq n\right)$. Finally, the perception probability of each sensor is integrated, and the time cost is O(1). The total time complexity is $2\times O(n)+O(n^{\prime })+O(1)$. Taking the maximum order and ignoring the coefficient, the computational complexity of coverage is O(n).

### 4.3. Full Coverage Analysis

**Figure 7:**
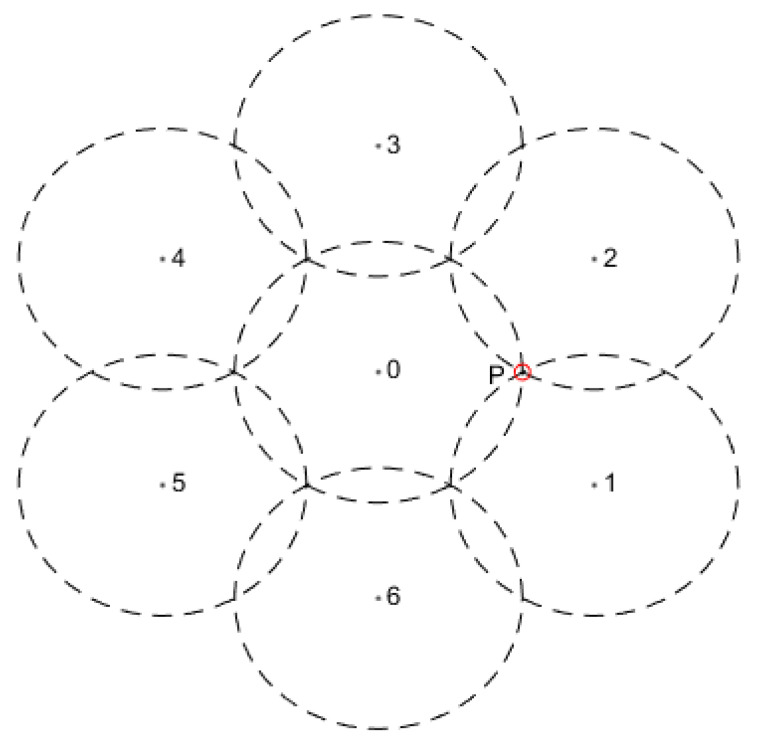
The tessellation structure.

Full coverage is a type of area coverage aimed at achieving effective perception across the entire region. From a structural perspective, full coverage can be represented as a tessellation problem in two-dimensional space. The optimal tessellation pattern for maximizing the utilization of a sensor’s sensing range is a regular hexagonal tessellation. The structure of this tessellation and the resulting coverage pattern are illustrated in Figure 7.

The 0/1 perception model is often used to study full coverage due to its simplicity in describing the interactions between multiple sensor coverage areas. In the 0/1 perception model, full coverage following the tessellation structure shown in Figure 7 is considered optimal for minimizing redundant sensor coverage. However, in the proposed perception model based on path loss and false alarm probability, the actual perception probability of sensors in WSNs does not drop immediately to zero at the defined sensing radius $r_{s}$, it gradually decays beyond this radius.

Under the joint perception of multiple sensors, the peripheral sensor nodes (nodes 1–6 in Figure 7) can be positioned slightly farther from the central node (node 0) while still ensuring full area coverage. According to the principles of the Voronoi diagram, the perception probability at the boundaries between sensor coverage areas is minimized, such as at point P in Figure 7. This point is equidistant from the three closest sensor nodes (0, 1, and 2), with this distance being the defined sensing radius $r_{s}$ in the 0/1 model.

Considering the perception effect of the three nearest nodes, the joint perception probability at point P, based on Equation (32), is given by

(36) $$p_{\mathrm{cov}}(s_{0,1,2},P)=1-{\left\{\Phi \left[\Phi^{-1}\left(1-\alpha \right)-\frac{P_{t}G_{t}G_{r}\lambda^{2}}{\sigma {\left(4\pi \right)}^{2}L}\cdot \frac{d_{0}^{\eta -2}}{d^{\eta }}\right]\right\}}^{3}$$

From the above equation, it can be determined that, given an acceptable perception probability ε, the distance between point P and the sensor is

(37) $$d={\left\{\frac{P_{t}G_{t}G_{r}\lambda^{2}d_{0}^{\eta -2}}{\sigma {\left(4\pi \right)}^{2}L\left[\Phi^{-1}\left(1-\alpha \right)-\Phi^{-1}\left(\sqrt[{3}]{1-\varepsilon }\right)\right]}\right\}}^{1/\eta }$$

Under the parameters in Table 2, it can be determined that d=7.9864 is greater than the defined sensing radius $r_{s}$ in the 0/1 perception model. Additionally, sensor nodes located at greater distances, such as nodes 3 to 6, which lie within the maximum sensing radius R from point P, can still contribute to the perception probability at this point. This allows for an increase in the relative distance between sensors, enabling the proposed probabilistic perception model to achieve a larger effective coverage area with the same number of sensors.

## 5. Simulation Testing

This section presents simulation testing to verify the feasibility of the proposed solution. The experimental hardware environment includes an AMD Ryzen 7 4800H CPU, 16 GB RAM, Windows 11 operating system, and MATLAB R2018a as the testing platform.

The objective of WSN node coverage optimization is to maximize effective coverage when the number of sensors is insufficient to fully cover the entire area. To address this, an optimization algorithm is employed to iteratively determine the optimal positions of sensor nodes to maximize coverage.

**Figure 8:**
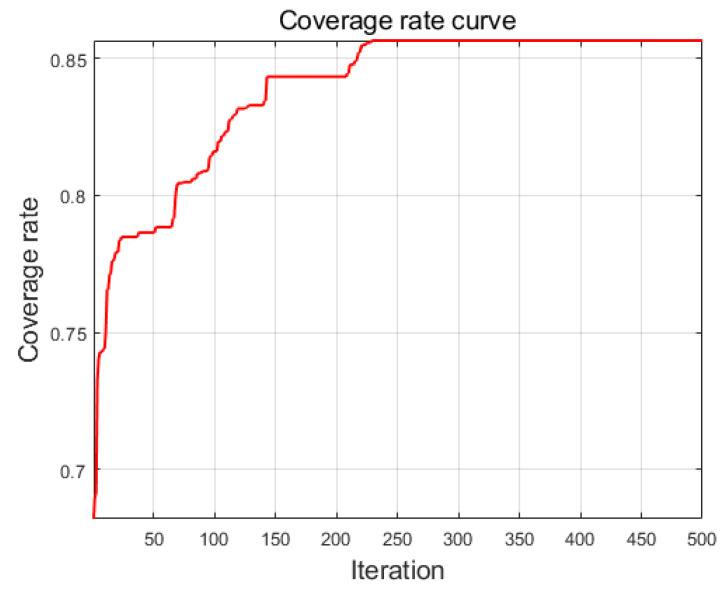
The coverage rate curve.

The parameters for the perception model are configured according to Table 2, with a satisfactory joint perception probability ε=0.8. The test deploys 15 sensor nodes within a range of 50×50, and the area coverage rate is calculated using Equation (35). The coverage rate curve generated by the intelligent algorithm is shown in Figure 8.

**Figure 9:**
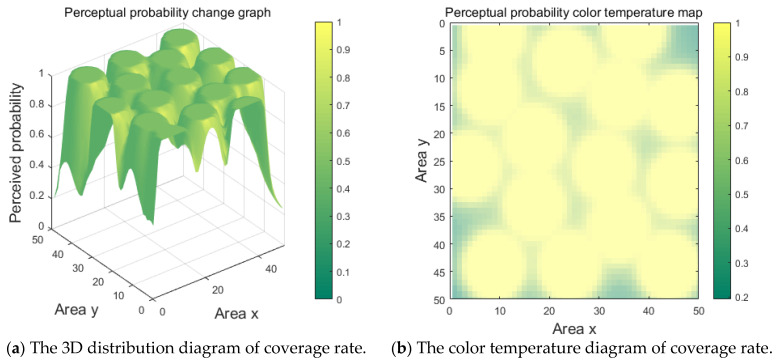

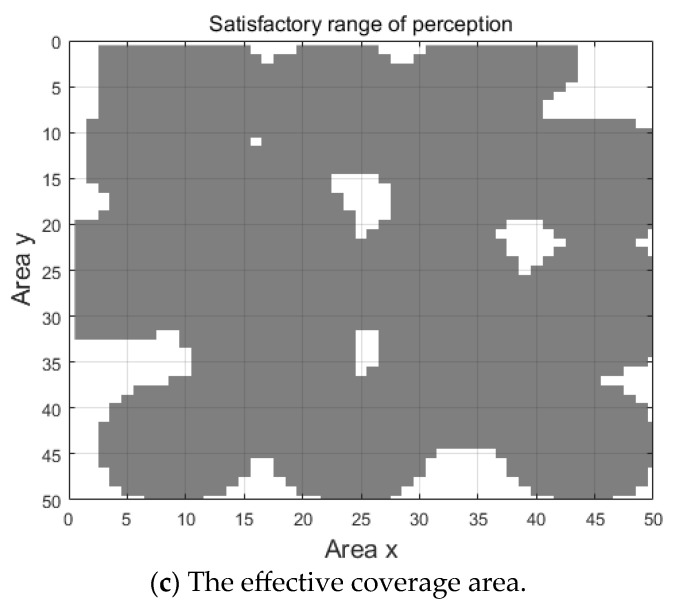
The coverage rate distribution and satisfactory coverage area.

The coverage rate distribution is illustrated in Figure 9a,b, while the satisfactory coverage area that meets the perception probability requirements is shown in Figure 9c.

Figure 9a is the 3D distribution diagram of regional coverage, and Figure 9b is the color temperature diagram of coverage rate. In order to better show the situation of coverage rate, only the regions that meet the coverage requirements are dyed, and the coverage situation is shown in Figure 9c. According to the calculation of the proportion of the dyed part in the figure, it is the regional coverage rate. According to Equation (35), the coverage rate of the experiment is 86.04%.

**Figure 10:**
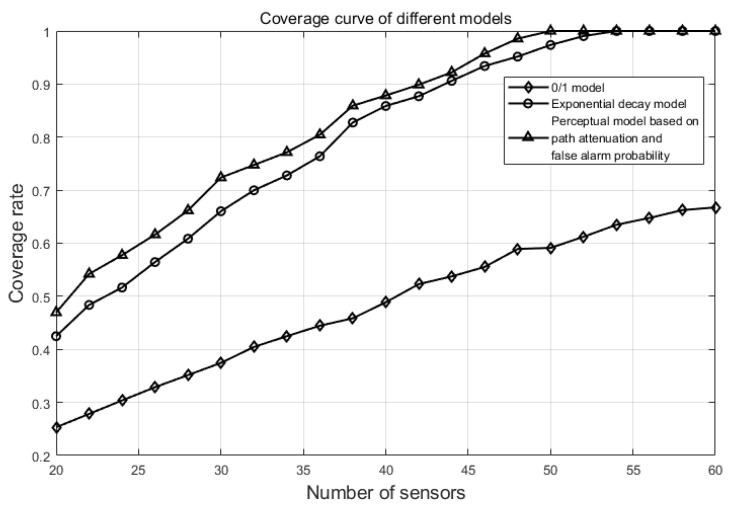
The coverage rate increases under different models.

The test evaluates coverage over areas using the 0/1 perception model, the exponential decay perception model, and the perception model based on path loss and false alarm probability. The number of coverage nodes is varied from 20 to 60. The experimental environment, coverage conditions, and optimization algorithms adopted in the test are the same. The regional coverage rate is obtained by Formula (33), and the difference only lies in the different formula of perception probability $p_{\mathrm{cov}}$ of the target point substituted by different perception models. For the 0/1 perception model, the exponential decay perception model, and the perception model proposed in this paper, Formula (1), Formula (2), and Formula (30) are used, respectively. The resulting change in area coverage rate is shown in Figure 10.

With 20 nodes, the coverage rate using the path loss and false alarm probability-based perception model is 46.95%, compared to 25.31% and 42.48% for the 0/1 perception model and exponential decay model, respectively. When the number of nodes is 50, the path loss and false alarm probability-based model is the first to achieve 100% coverage, while the exponential model reaches 100% coverage at 54 nodes. Even with a maximum of 60 sensor nodes, the 0/1 model only achieves a 66.75% coverage rate. Clearly, the proposed model achieves a larger area coverage rate compared to the 0/1 and exponential decay models with the same number of nodes, thereby reducing the number of nodes needed for coverage.

## 6. Conclusions

This paper presents an optimized coverage strategy for wireless sensor network (WSN) nodes based on path loss and false alarm probability. The innovation of this model lies in the combination of the path attenuation model of non-line-of-sight transmission of signals and the situation of misjudgment and failure of sensors in signal detection. This model can better describe the real detection situation of sensors in complex working environments and provide an excellent solution for improving WSN coverage performance and saving coverage costs. First, a probabilistic sensor perception model incorporating path loss and false alarm rate was established using a path loss model for signals and the Neyman–Pearson criterion from classical signal detection. Second, the effects of key parameters in the perception model on detection probability were analyzed, and a formula for the sensing radius was derived. Third, an objective function for the optimization problem was constructed based on the joint perception probability of multiple sensors. Finally, an intelligent optimization algorithm was employed to solve the objective function and validate the effectiveness of the proposed solution.

Through simulation experiments, the coverage of different sensing models is compared under the same coverage area. With the increase in the number of coverage nodes, the model proposed in this paper can achieve complete coverage of the entire area when the number of sensors is 50, while the exponential attenuation model requires 54, and the coverage rate of the 0/1 model is only 66.75% when the number of nodes is up to 60. According to the results of simulated experiments, the perception model based on path loss and false alarm probability developed in this study more accurately reflects the actual sensing capabilities of sensors in WSNs. This model achieves a larger effective coverage area with the same number of sensor nodes, thereby reducing the number of nodes required and enhancing network performance and longevity.

Due to limitations in the research environment, this study demonstrates the practical value of the proposed model and solution primarily through simulation experiments and theoretical analysis. However, testing in real-world application scenarios remains limited. Further research should focus on tests in the actual environment and further improve the model parameters through the comparison of test results in the actual environment, so as to better meet the needs of engineering applications.

## Figures and Tables

**Table 1:** Path Loss Exponents in Different Environments.

Environment Type	Path Loss Exponent η
Free Space	2
Urban Cellular	2.7~3.5
Urban Cellular with Shadowing	3~5
Indoor Short-Distance Line-of-Sight	1.6~1.8
Indoor Non-Line-of-Sight	4~6
Factory Non-Line-of-Sight	2~3

**Table 2:** Parameter Settings.

Parameter Name	Value
Transmit Power	$P_{t}=1$
Transmit Gain	$G_{t}=1$
Receive Gain	$G_{r}=1$
Carrier Wavelength	λ=100
System Hardware Loss	L=1
Reference Distance	$d_{0}=5$
Minimum Detectable Power	$P_{r0}=0.1$
False Alarm Rate	α=0.05 (Default value)
Standard Deviation	σ=0.2 (Default value)
Path Loss Coefficient	η=2.7 (Default value)

## Data Availability

Data are contained within the article.
